# Editorial: Functional columnar organization and long-range circuits in different cortical systems

**DOI:** 10.3389/fnsys.2023.1168606

**Published:** 2023-03-20

**Authors:** Kerstin E. Schmidt, Ralf A. W. Galuske

**Affiliations:** ^1^Brain Institute, Federal University of Rio Grande do Norte, Natal, Rio Grande do Norte, Brazil; ^2^Göttingen Campus Institute for Dynamics of Biological Networks, University of Göttingen, Göttingen, Germany; ^3^Department of Biology, Darmstadt University of Technology, Darmstadt, Hesse, Germany; ^4^Centre for Cognitive Science, Darmstadt University of Technology, Darmstadt, Hesse, Germany

**Keywords:** long-range horizontal intrinsic connection, cortical map, patchy activation, functional connectivity, cortical column network, cortical columns, cortico-cortical axons

The functional column is a classical concept which was introduced in the last century. This concept puts columns as organizing cortical units. It has largely influenced practical and theoretical research and also our understanding of neocortical processing. While it was initially attributed to early sensory cortical areas such as the primary visual cortex it can also be identified in higher sensory, multimodal or motor areas and their connections. Concomitant with the clustering of neurons of similar functional characteristics into periodic domains or columns extending through many cortical layers came the notion of a network of biased horizontal axons preferentially linking neurons with similar functional properties over short and long distances within a cortical area, and even between cortical areas, in a patchy manner. However, there is ample evidence for both selective and unselective long-range axons questioning the functional role of patchiness and the concept of the functional column in general.

Two recent opinions published in Frontiers of Systems Neuroscience highlight some of the diverging views on the topography of long-range cortical circuits. Whereas, Rockland points out a (functional) variety in connectivity rules as reason for the heterogeneity of termination patterns of long-range axons, Barbas et al. emphasize the systematic structural variation of cortical circuits which emerges because of timing differences in the phylogeny of the distinct areas. According to this interpretation, both horizontal laminar and vertical columnar types of termination patterns in the brain follow relational architectonic rather than functional rules. Laminar (horizontal) terminals, prevalent in feedback, could constitute a more ancestral pattern in which columnar patterns interconnecting areas with similar laminar profiles across different cortical systems (feedforward and horizontal) were inserted subsequently. The variety of module sizes would be explained by intersection constraints (convergence) of laminar and vertical patterns at different phylogenetic and developmental brain stages (Schmidt et al., 1999). This would be compatible with the known increase of module size and of complexity/contingency of represented features in high-level areas (e.g., Hutsler and Galuske, 2003). Conformingly, color sensitive patches of squirrel monkey have been recently demonstrated to increase in size and represent true hue-sensitivity, as an example for a more recently acquired emerging visual function of trichromatic primates, depending less and less on exact low level features such as cone opponency as visual hierarchy proceeds (Du et al.).

Apart from the notion that non-patchy and patchy termination patterns might have emerged at different phylogenetic stages, both, Rockland and Barbas et al. acknowledge that their co-existence eventually stands for distinct modes of communication. This is supported by the observation that different feedback, lateral and feedforward circuits seem to occupy not only different spatial, but also different temporal domains (Bastos et al., 2015; Vezoli et al., 2021). In recent original work of reconstructing single cell axons and their bouton distribution in the squirrel monkey's somatosensory cortex, Mir et al. came to the conclusion that axonal patches are termination fields of long-distance lateral connections and constitute special cortical loci of axonal convergence which attract axons of a population of pyramidal neurons nearby or based on the fact that they share functional properties with the presynaptic neurons. They assign different functional and parallel roles for patch and non-patch regions and different velocities of axons inside and outside. In accordance with the idea of phylogeny, patchy connections are seen as “characteristic wiring motif of the cerebral networks of higher order mammals” (Roe, 2019). This is significant given that apparently not all mammal lineages express functional columns (Schmidt and Wolf, 2021) and patchy intrinsic connections (e.g., Van Hooser et al., 2006).

Rockland (Martin et al., 2014; Chavane et al., 2022; Innocenti et al., 2022) argue that structure and function of lateral intra- and cortico-cortical connections in the different parallel and hierarchically arranged cortical systems might be far too heterogeneous to define a common motif. However, we assume that the connectional biases expressed in patchiness and columnar layouts are too striking, too exuberant and probably too costly in implementation to lack any functional meaning and advantage. We propose that this circuitry enables fast selection of circuits facilitating the grouping of similar features for further joint processing, and the application and constant actualization of behaviorally relevant simple predictions such as known in the visual system for Gestalt criteria (e.g., Schmidt et al., 1997). Eventually, comparative studies of the behavioral strategies of mammals adopting larger scale and repetitive layouts of columns of functionally similar neurons with those expressing no such layouts might shed light into physiological significance and behavioral consequences of patch and non-patch structures in long-range connections.

## Author contributions

Both authors listed have made a substantial, direct, and intellectual contribution to the work and approved it for publication.
